# Comparative data of molecular weight distribution of agave fructans fractions using MALDI-ToF and HPLC-SEC

**DOI:** 10.1016/j.dib.2019.103984

**Published:** 2019-05-10

**Authors:** Lorena Moreno-Vilet, Stéphane Bostyn, José-Luis Flores-Montaño, Rosa-María Camacho-Ruiz

**Affiliations:** a CONACYT-CIATEJ-Centro de Investigación y Desarrollo en Agrobiotecnología Alimentaria. Pachuca Ciudad del Conocimiento y la Cultura. Boulevard Circuito La Concepción 3 C.P.42162. San Agustín Tlaxiaca, Hgo; b Centro de Investigación y Asistencia en Tecnología y Diseño del Estado de Jalisco A.C., Av. Normalistas 800, Colinas de la Normal, C.P. 44270, Guadalajara, Jalisco, Mexico; c Institut Universitaire de Technologie, Université d’Orléans, 16 rue d’Issoudun, BP 16729- 45067 Orléans, Cedex 2, France

## Abstract

A comparative data of two generally accepted mass techniques for estimate the molecular weight distribution of polysaccharides are presented. The data were obtained from agave fructans samples of different molecular weight, which were analyzed authored independently. The two analytical techniques used were Matrix-Assisted Laser Desorption/Ionization Time-Of-Flight Mass Spectrometry (MALDI-ToF) and High Pressure Size Exclusion Chromatography (HP-SEC). The data set here are related to the research paper entitled “Size-exclusion chromatography (HPLC-SEC) technique optimization by simplex method to estimate molecular weight distribution of agave fructans” by Moreno-Vilet et al. [1]. This article present the comparative figures as histograms obtained from mathematically processed MALDI-ToF spectra and HPLC-SEC chromatograms. And also, the calculated polymer parameters as number and mass molecular weight, number and mass average degree of polymerization and dispersity index.

**Table:** Specifications table

Subject área	*Chemistry*
More specific subject área	*Molecular weight distribution of polysaccharides by two techniques*
Type of data	*Table and figures*
How data was acquired	*Agave fructans samples of different molecular weight obtained commercially and previously fractionated in laboratory.* *The HPLC-SEC system consisted of a* 1220 *Infinity LC System HPLC coupled with a refractive index detector (Agilent, Alpharetta GA, USA). An Ultrahydrogel DP column and guard column (7.*8 mm d*.i. ×* 300 mm*, Waters, Milford, MA, USA) was used as stationary phase. The mobile phase was prepared with deionized and bidistilled water.* *MALDI-ToF-MS: UltraFlex I mass spectrometer (Bruker Daltonics) equipped with a* 337 nm *nitrogen laser and a grid-less delayed extraction ion source.*
Data format	*Analyzed*
Experimental factors	*Commercial and laboratory extracted and purified agave fructans in powder*
Experimental features	*Comparison between two mass analytical techniques to determine the size distribution of agave fructans.*
Data source location	*Agave fructans of Agave tequilana Webber var. azul from Jalisco, México*
Data accessibility	*Data is provided with this article*
Related research article	Moreno-Vilet, L., Bostyn, S., Flores-Montaño, J.-L., & Camacho-Ruiz, R.-M. (2017). Size-exclusion chromatography (HPLC-SEC) technique optimization by simplex method to estimate molecular weight distribution of agave fructans. *Food Chemistry*, *237*[1].

**Table:** 

**Value of the data** • Comparison between two mass analytical techniques, one accessible and cheap (HPLC-SEC) and the other expensive (MALDI-ToF), is carried out in qualitative and quantitative analysis. • Data from agave fructans fractions related with polymer size parameters are presented, it is known that biological and technological effects of fructans depend on these parameters. • The data provide the range of polymerization degree where both techniques are equivalent. • Data related with typical polymer parameters and histograms from Agave tequilana fructans can be compared with other agave species or different fructans sources. • Data can be used by researchers performing analysis of agave fructans, related with their characterization or their effects on health, and by developers of new products containing agave fructans.

## 1 Data

**Fig. 1:**
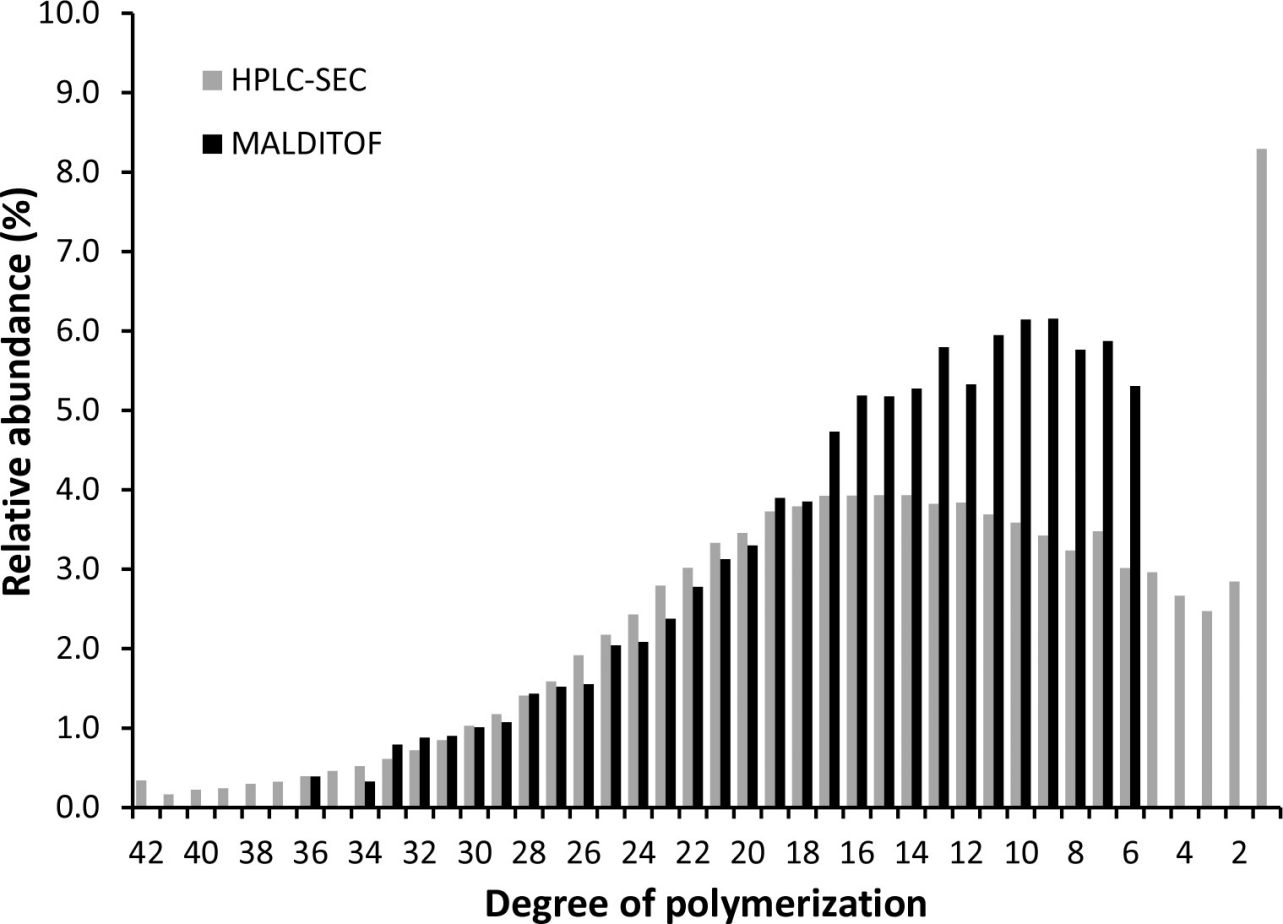
Histograms of size distribution of commercial agave fructans obtained from MALDI-ToF-MS spectra and HPSLC-SEC chromatogram. Differential Molecular Weight Distribution: molar fractions normalized to area = 100%.

**Fig. 2:**
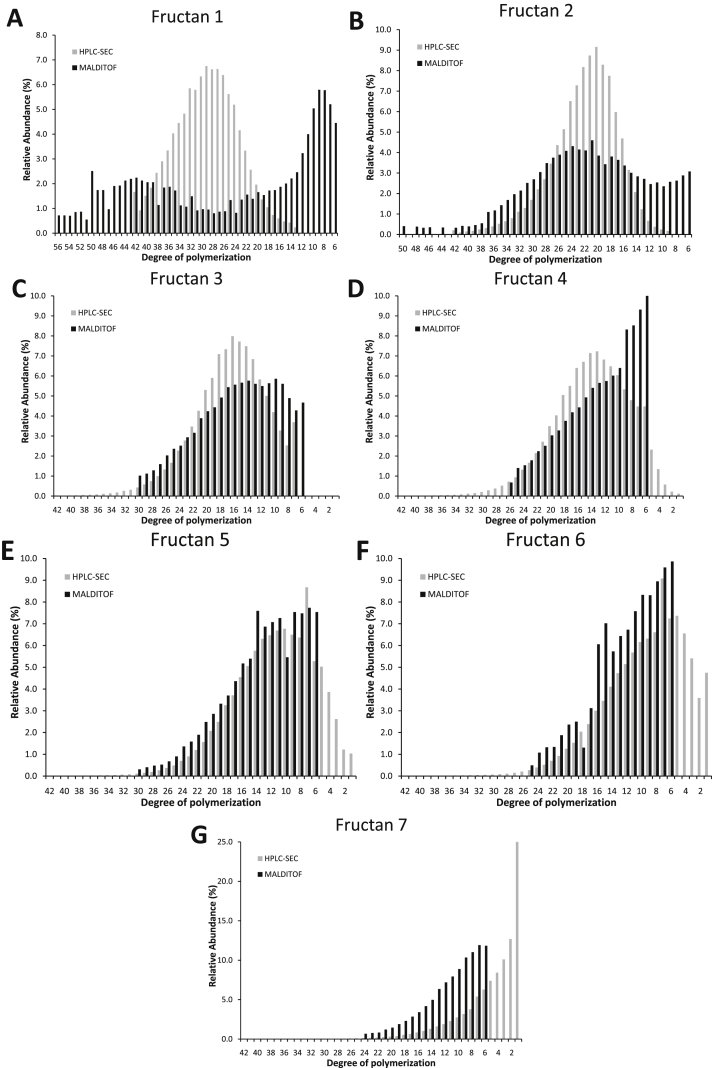
Histograms of size distribution of different agave fructans fractions obtained from MALDI-ToF-MS spectra and HPSLC-SEC chromatogram. A) fructan 1, B) fructan 2, C) fructan 3, D) fructan 4, E) fructan 5, F) fructan 6, G) fructan 7. Differential Molecular Weight Distribution: molar fractions normalized to area = 100%.

Fig. 1, Fig. 2 are the comparison of histograms obtained by HPLC-SEC and MALDI-ToF-MS, authored independently for the different samples. MALDI-ToF histogram starts in DP 6 (due to the acquisition mode), but has similar shape of DP ranging from DP 6 to 36 while HPLC-SEC ranging from DP 1 to 42. The first one is based on soft ionization of molecules to produce a mass spectra where can be identified a peak for each DP, while HP-SEC is based on size exclusion dependent of hydrodynamic volume of molecules.

To compare both techniques in numerical terms, the MALDI-ToF high mass spectra and HP-SEC chromatogram were mathematically processed as proposed by Mané [2] and Moreno-Vilet [1] respectively. Thus, the molecular weight distributions of the different samples of fructans were calculated by the classical polymer parameters. Table 1 shows the processed data for each sample analyzed by both techniques. The samples of higher molecular weight (samples 1 and 2) have greater differences in the calculated parameters and size distribution in the histograms between both techniques. Within the range of a DP 6 to 42, both techniques are equivalent.

**Table 1:** Comparative data of molecular weight parameters of different agave fructans samples obtained by MALDI-ToF-MS and HPLC-SEC.

	MALDI-ToF-MS					HPLC-SEC				
Sample	Mn (Da)	Mw (Da)	D_Pn_	DP_w_	D	Mn (Da)	Mw (Da)	DP_n_	DP_w_	D
Comercial	2510.81^a^	3004.49^a^	15.38^a^	18.42^a^	**1.20**^a^	2446.76^a^	3381.74^a^	14.98^a^	20.75^a^	**1.38**^**b**^
1	**4158.82**^**a**^	**5769.76**^**a**^	**25.54**^**a**^	**35.48**^**a**^	**1.39**^**a**^	**4653.71**^**b**^	**4837.05**^**b**^	**28.60**^**b**^	**29.73**^**b**^	**1.04**^**b**^
2	3552.77^a^	**4224.82**^**a**^	21.81^a^	**25.95**^**a**^	**1.19**^**a**^	3544.15^a^	**3709.34**^**b**^	21.75^a^	**22.77**^**b**^	**1.05**^**b**^
3	2523.12^a^	2908.77^a^	15.45^a^	17.83^a^	1.15^a^	2710.66^a^	2961.26^a^	16.61^a^	18.16^a^	1.09^a^
4	2062.33^a^	**2419.42**^**a**^	12.61^a^	**14.81**^**a**^	1.17^a^	2313.51^a^	**2641.50**^**b**^	14.16^a^	**16.18**^**b**^	1.14^a^
5	2156.53^a^	2500.28^a^	13.19^a^	15.31^a^	1.16^a^	1933.82^a^	2342.87^a^	11.82^a^	14.34^a^	1.21^a^
6	**1955.81**^**a**^	2241.60^a^	11.95^a^	13.72^a^	**1.15**^**a**^	**1581.67**^**b**^	2092.02^a^	9.65^a^	12.79^a^	**1.32**^**b**^
7	**1800.79**^**a**^	2058.24^a^	**11.00**^**a**^	12.59^a^	1.14^a^	**832.7**^**b**^	1514.56^a^	**5.03**^**b**^	9.23^a^	1.82^a^

**Mn**: number average molecular weight, **Mw**: weight average molecular weight, **DP**_**n**_: number average degree of polymerization, **DP**_**w**_**:** weight average degree of polymerization, **D:** Dispersity index. Values with same letter indicate no statistically significant differences between methods, *t*-test (p < 0.05).

## 2 Experimental design, materials and methods

### 2.1 Materials

Agave fructans (from *A. tequilana Weber var. azul*) Olifructine ^®^ from Nutriagaves de México S.A. de C.V. and agave fructans of different molecular weights obtained from CIATEJ laboratory.

### 2.2 High Performance Liquid Size-Exclusion Chromatography (HPLC-SEC)

Samples were diluted in deionized and bidistilled water at 5 gL^−1^ and passed through a 0.45μm polyethersulfone (PES) membrane, 15 mm diameter, syringe filter (Agilent, CA, USA). The HPLC-SEC system consisted of a 1220 Infinity LC System HPLC coupled with a refractive index detector (Agilent, Alpharetta GA, USA). An Ultrahydrogel DP column and guard column (7.8 mm d.i. × 300 mm, Waters, Milford, MA, USA) was used as stationary phase, with size exclusion range from 100 to 5000 Da. The volume of the injected sample was 50 μL. As a mobile phase, deionized and bidistilled water was used at pH of 5.4, adjusted with NaOH or HCl, it was degassed and filtered through a 0.45μm PES membrane. It was operated at 0.36 mL/min and 61.7 °C of column temperature. The system was calibrated with molecular weight standards: dextrans of 1000 Da, maltoheptaose DP7, maltohexaose DP6, maltopentaose DP5, maltotetraose DP4, nystose, 1-kestose, sucrose and fructose. ChemStation with Agilent OpenLAB software were used for controlling the system operation and data analysis, according to the methodology described by Moreno et al. [1].

### 2.3 Matrix-Assisted Laser Desorption/Ionization Time-Of-Flight Mass Spectrometry (MALDI-ToF-MS)

MALDI-ToF-MS spectra were carried out on an UltraFlex I mass spectrometer (Bruker Daltonics) equipped with a 337 nm nitrogen laser and a grid-less delayed extraction ion source. Samples were dissolved in water in the mg/mL range and the matrix 2,5-dihydroxybenzoic acid was prepared at 10 mg/mL in a solution of 1:1 methanol:water. Samples (0.5 μL) and matrix solution were spotted and air-dried on a stainless-steel plate. Spectra were acquired in the linear positive mode (200 laser shots) in the *m*/*z* range from 800 to 20 000 and in the reflectron positive mode (200 laser shots) in the *m*/*z* range from 50 to 5000. A pepmix calibration kit (Bruker Daltonics) and apomyoglobin were used for calibration. The instrument was controlled using Flex Control 3.3 software (Bruker Daltonics) and spectra were processed using FlexAnalysis 3.3 software from Bruker Daltonics. Two acquisitions were performed for high and low mass (Linear and reflectron mode) in order to improve visualization of higher DP peaks without interference from lower mass peaks that can dominate the spectra if they are included in the same acquisition.

### 2.4 Estimation of molecular weight distribution

The molecular weight distributions of the different unknown samples of agave fructans were calculated by the classical polymer parameters by the following equations:

Number-average molecular weight:

[1] $${\bar{M}}_{n}=\frac{\sum n_{\text{i}}{\text{M}}_{\text{i}}}{\sum n_{\text{i}}}$$

Weight-average molecular weight:

[2] $${\bar{M}}_{w}=\frac{\sum n_{i}{\text{M}}_{\text{i}}^{2}}{\sum n_{i}{\text{M}}_{\text{i}}}$$

where *n* is the differential area under the curve in the HP-SEC chromatogram or absolute intensity (*I*) of the MALDI-ToF spectra and *M* the molecular weight of the fraction *i*.

Dispersity index:

[3] $$D=\frac{M_{w}}{M_{n}}$$

Number and weight average degree of polymerization of fructans:

[4] $${\bar{DP}}_{n}=\frac{M_{n}-18}{162.1}\;\;\;\;\;\;{\bar{DP}}_{w}=\frac{M_{w}-18}{162.1}$$
