# Supplementary material for: Comparative data of molecular weight distribution of agave fructans fractions using MALDI-ToF and HPLC-SEC
Source: Data Brief. 2019 May 10;24:103984. doi: 10.1016/j.dib.2019.103984 (PMC6529771; doi:10.1016/j.dib.2019.103984)
Supplement: Multimedia component 1 [file mmc1.docx]

**Editorial office**

**Data in Brief**

Regarding our manuscript entitled “**Comparative data of molecular weight distribution of agave fructans fractions using MALDI-ToF and HPLC-SEC**”, we confirm that all the listed authors do not have any possible conflicts of interest.

We look forward to hearing favorable news from your side**.**

Kind regards,

Rosa María Camacho, D.Sc.

Researcher, CIATEJ

rcamacho@ciatej.mx
